# Transcriptome Analysis Reveals Sertoli Cells Adapting Through Redox and Metabolic Pathways Under Heat Stress in Goats

**DOI:** 10.3390/genes15121582

**Published:** 2024-12-09

**Authors:** Guang Yang, Yiwei Wang, Pengyun Ji, Bingyuan Wang, Guoshi Liu

**Affiliations:** 1 College of Animal Science and Technology, Sanya Institute of China Agricultural University, Sanya 572025, China; yangguangimu@163.com; 2 State Key Laboratory of Farm Animal Biotech Breeding, Frontiers Science Center for Molecular Design Breeding, College of Animal Science and Technology, China Agricultural University, Beijing 100193, China; s20233040771@cau.edu.cn (Y.W.); jipengyun@cau.edu.cn (P.J.); wangbingyuan@cau.edu.cn (B.W.)

**Keywords:** goat, Sertoli cell, autophagy, oxidative stress, transcriptome analysis

## Abstract

Background/Objectives: Climate change-induced temperature elevations pose significant challenges to livestock reproduction, particularly affecting testicular function in small ruminants. This study investigates the acute heat-stress response in goat Sertoli cells (SCs), aiming to elucidate the molecular mechanisms underlying heat-induced damage to male reproductive tissues. Methods: SCs were isolated from testes of 4-month-old black goats and exposed to heat stress (44 °C for 2.5 h). We employed transcriptome sequencing, CCK-8 assay, electron microscopy, ROS measurement, autophagy detection, Western blot analysis, and lactate concentration measurement. Bioinformatics analyses including Gene Ontology (GO), Kyoto Encyclopedia of Genes and Genomes (KEGG) pathway, and protein–protein interaction network analyses were performed on the transcriptome data. Results: Heat stress significantly reduced SC viability, induced oxidative stress and autophagy, and altered gene expression profiles. We identified 1231 significantly differentially expressed genes, with significant enrichment in membrane-related processes and metabolic pathways. Metabolism-related genes, including *PKLR*, *ACOT11*, and *LPCT12*, were significantly downregulated. Protein–protein interaction network analysis revealed ten hub genes potentially crucial in the heat-stress response: *HSP90AA1*, *HSPA5*, *BAG3*, *IGF1*, *HSPH1*, *IL1A*, *CCL2*, *CXCL10*, *ALB*, and *CALML4*. Conclusions: This study provides comprehensive insights into the molecular mechanisms underlying goat SC response to heat stress. The identified genes and pathways, particularly those related to metabolism and stress response, offer potential targets for developing strategies to mitigate heat-stress effects on livestock reproduction. These findings contribute to our understanding of climate change impacts on animal husbandry and may inform the development of heat-stress resistant livestock lines.

## 1. Introduction

Global climate change has led to increasing temperatures and extreme weather events, posing a substantial threat to animal reproductive health, particularly in livestock [1,2,3]. According to the Intergovernmental Panel on Climate Change, global surface temperature has risen by approximately 1 °C since pre-industrial times, with further increases projected [4,5,6]. Goats, economically significant livestock, are highly susceptible to heat stress, which can impair their reproductive capabilities [7,8,9].

The testes, particularly their constituent Sertoli cells (SCs), are especially vulnerable to heat-induced damage [10,11,12]. SCs play crucial roles in providing structural support, forming the blood-testis barrier, and regulating the microenvironment essential for spermatogenesis [13,14]. Heat stress can disrupt these functions, leading to impaired spermatogenesis and reduced reproductive capacity [15,16]. Despite the importance of maintaining SC function under heat stress, research on the effects of high temperatures on goat testicular supporting cells remains limited [17,18].

To address this knowledge gap, our study employs transcriptome sequencing to examine the molecular responses of goat SCs to acute heat stress (44 °C for 2.5 h). This approach allows us to analyze gene expression changes, identify key molecular pathways, and discover potential biomarkers for heat-stress damage [19].

The objectives of this study are to elucidate the physiological changes in SCs under heat stress, identify key genes and pathways involved, explore potential protective strategies, and provide novel insights to aid the livestock industry in adapting to climate change challenges [14,20]. By focusing on goat SCs, this research fills a critical gap in understanding heat-stress effects on livestock reproduction. The findings have the potential to inform breeding strategies, develop heat-stress resistant livestock lines, and contribute to the sustainable development of the animal husbandry industry in the face of ongoing climate change.

## 2. Materials and Methods

### 2.1. SCs Isolation, Purification, and Verification

SCs were isolated from the testes of 4-month-old black goats sourced from a local farm (109°11′28.511″ E, 18°25′55.729″ N). Testicular tissue was enzymatically digested using collagenase IV and trypsin (Invitrogen, Carlsbad, NY, USA) at 32 °C. The resulting cell suspension was filtered through a 70 μm nylon mesh, centrifuged at 1000× *g* for 5 min, and the pellet was resuspended in Dulbecco’s Modified Eagle Medium/Nutrient Mixture F-12 (DMEM/F12) medium (Invitrogen, Carlsbad, NY, USA) supplemented with 10% fetal bovine serum. Cells were cultured at 32 °C in a humidified atmosphere containing 5% CO_2_. To ensure purity, the culture medium was changed every three days to remove residual germ cells. The identity and purity of SCs were confirmed through morphological observation under phase-contrast microscopy, hematoxylin and eosin (H&E) staining, Oil Red O staining for lipid droplets, and immunofluorescence staining for SC-specific markers WT1 and Vimentin.

### 2.2. Construction of an In Vitro Heat-Stress Model for SCs

To establish an in vitro heat-stress model for SCs, we systematically evaluated various temperature and duration combinations using the Cell Counting Kit-8 (CCK-8) assay (Servicebio, Wuhan, China). Isolated SCs were exposed to temperatures ranging from 32 °C to 44 °C (in 2 °C increments), with heat-stress durations of 0.5 to 3 h, followed by post-stress recovery periods of 0.5 to 3 h at 32 °C. Throughout the experiments, cells were maintained in a 5% CO_2_ environment. This optimization process aimed to identify conditions that induce significant stress responses while maintaining sufficient cell viability for subsequent analyses. Following each treatment condition, cell viability was evaluated using the CCK-8 assay to assess the impact of different heat-stress parameters and determine the optimal conditions for our model.

### 2.3. Transmission Electron Microscopy Imaging and Analysis

Collect cell pellets from heat-stressed and control (32 °C) cultures. Fix with electron microscopy fixative, wash with phosphate buffer. Embed in agarose, post-fix with osmium tetroxide. Dehydrate through ethanol series and acetone. Infiltrate with embedding medium, polymerize. Section resin blocks using ultramicrotome, collect on copper grids. Stain with uranyl acetate and lead citrate. Observe under transmission electron microscope for image analysis.

### 2.4. Detection of Cellular Reactive Oxygen Species (ROS) and Autophagy Levels

For ROS measurement, cells (control and heat-stressed) were incubated with 10 μM 2′,7′-dichlorofluorescin diacetate (DCFH-DA) at 32 °C for 20 min, washed, and ROS levels were measured (excitation: 488 nm, emission: 525 nm). For autophagy detection, cells were stained with Monodansylcadaverine (MDC) solution at 32 °C for 30 min in the dark, washed with Assay Buffer, and fluorescence was measured (excitation: 335 nm, emission: 512 nm).

Western blot analysis was performed to detect p62 and LC3-I/LC3-II protein levels in control (32 °C) and heat-stressed SCs. Proteins were extracted, quantified, and separated by Sodium Dodecyl.

Sulfate-Polyacrylamide Gel Electrophoresis (SDS-PAGE) was carried out before the transfer to Polyvinylidene Difluoride (PVDF) membranes. PVDF membranes were blocked, then incubated with primary antibodies against p62 and LC3-I/LC3-II (Servicebio, Wuhan, China), followed by HRP-conjugated secondary antibodies. Protein bands were visualized using Enhanced Chemiluminescence (ECL) and imaged. Band intensities were quantified and normalized to β-actin (Servicebio, Wuhan, China).

### 2.5. Transcriptome Sequencing

Total RNA was extracted using TRIzol reagent (Thermo Fisher, 15596018). RNA quality was assessed using a NanoDrop ND-1000 spectrophotometer (NanoDrop, Wilmington, DE, USA) and Agilent Bioanalyzer 2100 (Agilent, Santa Clara, CA, USA). Samples with concentration > 50 ng/μL, RNA Integrity Number (RIN) > 7.0, and total RNA > 1 μg were selected. Poly(A) mRNA was isolated using oligo(dT) magnetic beads (Dynabeads Oligo (dT), cat.25-61005, Thermo Fisher, Waltham, MA, USA) and fragmented using NEBNext Magnesium RNA Fragmentation Module (cat.E6150S, USA). cDNA synthesis was performed using SuperScript II Reverse Transcriptase (Invitrogen, cat.1896649, Carlsbad, CA, USA) for first-strand synthesis, followed by *E. coli* DNA polymerase I (NEB, cat.m0209, Ipswich, MA, USA) and RNase H (NEB, cat.m0297, Ipswich, MA, USA) for second-strand synthesis. The resulting DNA was end-repaired, A-tailed, and ligated to adapters. After size selection and Uracil-DNA Glycosylase (UDG) (NEB, cat.m0280, Ipswich, MA, USA) digestion, PCR amplification was performed. The final strand-specific library had an average insert size of 300 ± 50 bp. Paired-end sequencing (PE150) was conducted on an Illumina NovaSeq 6000 platform (LC Bio Technology Co., Ltd. Hangzhou, China) following standard protocols.

### 2.6. RT-qPCR Analysis

Total RNA was extracted from control and heat-stressed SCs using TRIzol reagent (Servicebio, Wuhan, China), with the quality assessed via NanoDrop spectrophotometry (A260/A280 ratio between 1.8 and 2.0). cDNA was synthesized using the PrimeScript RT reagent Kit, and RT-qPCR was performed using the SYBR Premix Ex Taq II kit (Servicebio, Wuhan, China) on a LightCycler 480 II system. PCR conditions included initial denaturation at 95 °C for 30 s, followed by 40 cycles (95 °C for 5 s, 60 °C for 30 s). Melting-curve analysis verified amplification specificity, with all samples run in triplicate. Relative gene expression was calculated using the 2^−ΔΔCt^ method, with β-actin as the reference gene. The primers required for RT qPCR are listed in Table S1.

### 2.7. Detection of Lactate Levels

Lactate concentration in cell culture media was determined using the Cedex Bio Lactate Assay Kit (Roche, Basel, Switzerland). The Cedex Bio instrument was initialized, calibrated, and quality-controlled according to the manufacturer’s instructions. A 2 μL aliquot of cell culture medium was mixed with assay reagents as per the kit protocol. The sample was then measured using the instrument. Lactate concentration data were recorded and analyzed.

### 2.8. Statistical Analysis

All statistical analyses were performed using GraphPad Prism version 8.0.0 (GraphPad Software, Inc., San Diego, CA, USA). Data from cell proliferation assays, ROS probe fluorescence measurements, and MDA probe fluorescence levels were analyzed using this software. One-way analysis of variance (ANOVA) was used for multiple comparisons. All data in figures are presented as mean ± standard error (S.E.). Each experiment was performed in triplicate, and a *p*-value < 0.05 was considered statistically significant.

## 3. Results

### 3.1. Identification of SCs

**Figure 1:**
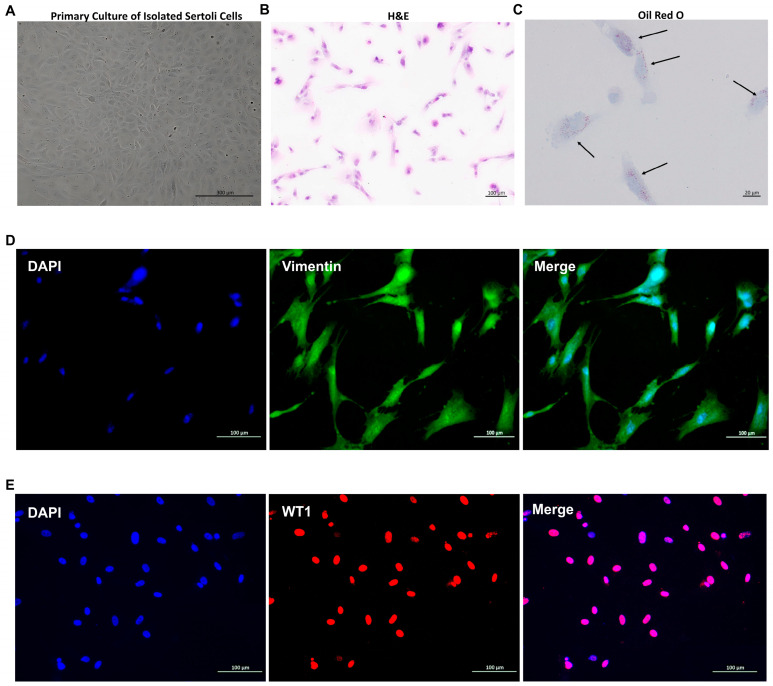
The isolation and identification of primary goat SCs. (**A**) Bright-field microscopy image of primary SCs. (**B**) H&E staining of primary SCs. (**C**) Oil Red O staining of primary SCs; black arrows indicate red lipid droplets. (**D**,**E**) Immunofluorescence staining of SC-specific proteins. (**D**) Vimentin (green fluorescence); (**E**) WT1 (red fluorescence). Cell nuclei were counterstained with DAPI (blue fluorescence). Scale bars are shown in the bottom right corner of each image.

Figure 1 illustrates the successful isolation and identification of SCs. Bright-field microscopy and H&E staining revealed cells exhibiting the typical morphology of SCs (Figure 1A,B). The presence of characteristic lipid droplets was confirmed through Oil Red O staining (Figure 1C). To further verify cell identity, immunofluorescence staining was performed for two specific SC markers: Vimentin (Figure 1D) and WT1 (Figure 1E).

### 3.2. Establishment of an In Vitro Heat-Stress Model for SCs

**Figure 2:**
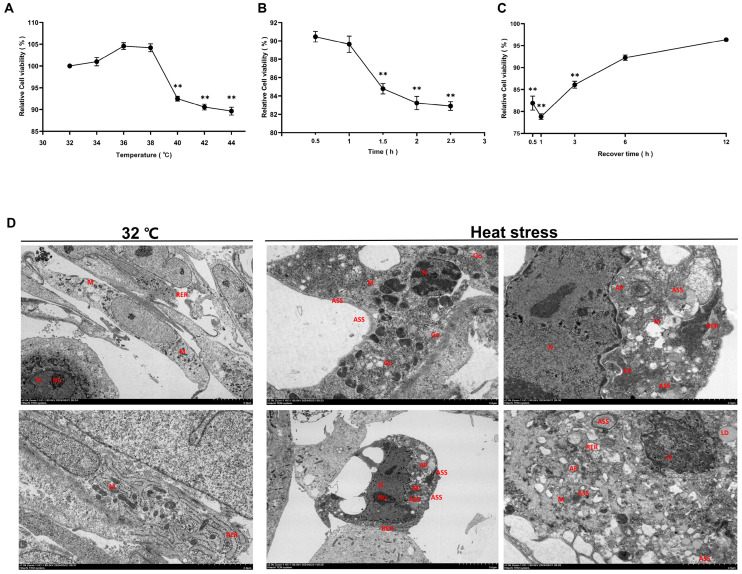
The effects of heat stress on goat testicular SC viability and ultrastructure. (**A**) CCK-8 analysis of SC viability at different temperatures. (**B**) The impact of different heat-stress durations on SC viability. (**C**) The effect of various recovery times on SC viability after heat stress. Data are presented as mean ± SEM (n = 6). ** *p* < 0.01 compared to control. (**D**) Transmission electron microscopy images of SCs before and after heat stress. Left: control group at 32 °C; right: heat-stress group at 44 °C. Scale bars are shown in the bottom right corner of each image. N: Nucleus; M: Mitochondria; RER: rough endoplasmic reticulum; LD: lipid droplets; ASS: autophagic lysosome; AP: autophagosome.

CCK-8 assay revealed that SC viability peaked at 36 °C (104.6% of 32 °C control) and decreased significantly above 38 °C, reaching a minimum at 44 °C (90% of control, *p* < 0.01) (Figure 2A). At 44 °C, viability declined gradually, reaching its lowest after 2.5 h (Figure 2B). During recovery at 32 °C, viability remained significantly lower for the first hour (*p* < 0.01) before gradually increasing (Figure 2C). Electron microscopy showed that heat-stressed SCs exhibited pronounced autophagic phenomena compared to controls at 32 °C, indicating significant subcellular responses to thermal stress (Figure 2D).

### 3.3. Identification of ROS and Autophagy Markers

**Figure 3:**
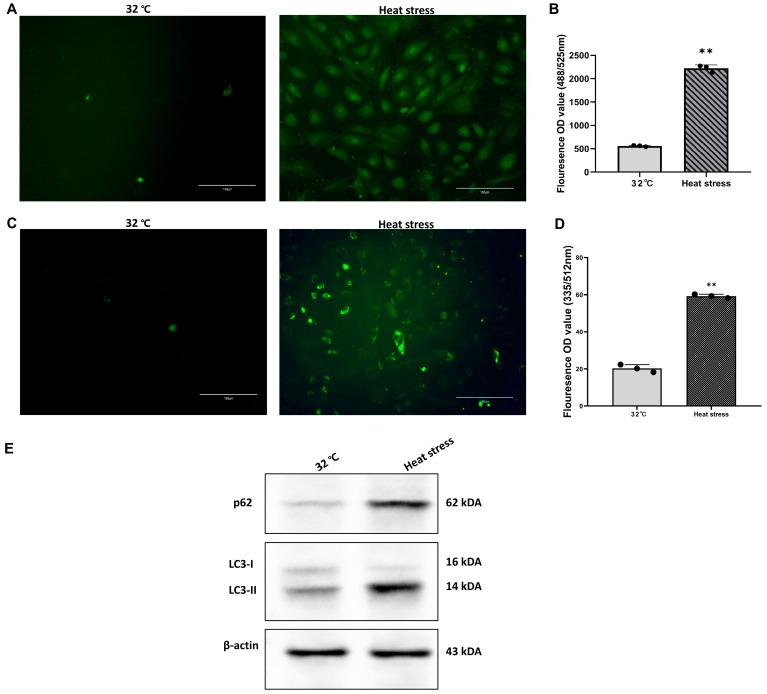
Heat stress induces ROS production and autophagy in goat testicular SCs. (**A**) Representative fluorescence images of ROS levels in SCs at 32 °C and after heat-stress treatment. Green fluorescence indicates DCFH-DA probe staining. Scale bars are shown in the bottom right corner of each image. (**B**) The quantification of relative fluorescence intensity (OD488/525 nm) in the control and heat-stress groups. ** *p* < 0.01. (**C**) Representative fluorescence images of autophagy levels in SCs at 32 °C and after heat-stress treatment. Green fluorescence indicates MDA probe staining. Scale bars are shown in the bottom right corner of each image. (**D**) The quantification of relative fluorescence intensity (OD335/512 nm) in the control and heat-stress groups. ** *p* < 0.01. (**E**) Western blot analysis of autophagy-related proteins p62 and LC3-I/LC3-II. β-actin was used as an internal reference.

We examined autophagy and ROS levels in SCs before and after heat stress (Figure 3A,C).We quantified the relative fluorescence levels representing ROS and autophagy in SCs under normal conditions and after heat-stress treatment (Figure 3B,D). Both significantly increased post stress, with ROS levels doubling and autophagy levels increasing 4-fold (*p* < 0.01). Western blot analysis (Figure 3E) showed an upregulation of autophagy markers p62 and LC3-I/LC3-II. The increase in LC3II expression indicates an increase in autophagosomes within the cell; the increase in P62 expression suggests that the degradation of autophagosomes is blocked.

### 3.4. Transcriptome Analysis of Heat-Stressed SCs

**Figure 4:**
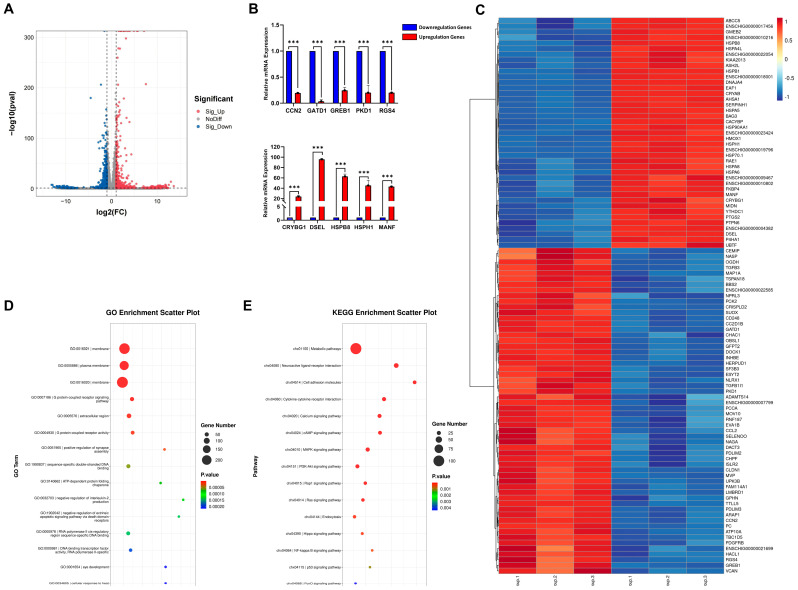
Heat stress induces gene expression changes and pathway enrichment in SCs. (**A**) A volcano plot of DEGs. Red dots represent upregulated genes in the heat−stress group, while blue dots represent downregulated genes. (**B**) RT−qPCR validation of DEGs. The bar graph shows the expression levels of 5 upregulated and 5 downregulated genes, randomly selected. *** *p* < 0.001. (**C**) A heatmap of the top 40 upregulated and 57 downregulated genes with the most significant expression changes. Color intensity indicates gene expression levels in the control and heat-stress groups. (**D**) GO enrichment analysis of DEGs. Shows the most significantly enriched biological processes, cellular components, and molecular functions. (**E**) KEGG pathway enrichment analysis of DEGs. Displays the most significantly enriched signaling pathways.

Transcriptomic analysis revealed significant transcriptional changes in heat-stressed SCs compared to controls (Table S2). We identified 1231 differentially expressed genes (DEGs) out of 17,440 total genes (fold change ≥ 1.5, *p* < 0.05), with 581 upregulated and 650 downregulated (Figure 4A). RT-qPCR validation of 10 randomly selected DEGs confirmed the consistency between these genes and the overall transcriptomic trends observed in the RNA-seq findings (Figure 4B). The top 40 upregulated and 57 downregulated genes are displayed in Figure 4C. Among the DEGs, we observed a significant upregulation of multiple inflammation-related genes following heat-stress treatment, including *PTGS2 (COX-2)*, *IL1A*, *CXCL10*, and *TLR9*, among others. Gene Ontology (GO) analysis (*p* < 0.05) revealed significant enrichment in processes such as “G protein-coupled receptor activity” (GO:0004930), “ATP-dependent protein folding chaperone” (GO:0140662), and “cellular response to heat” (GO:0034605) (Figure 4D). Kyoto Encyclopedia of Genes and Genomes (KEGG) pathway analysis showed enrichment in “Metabolic pathways” (chx01100), “PI3K-Akt signaling pathway” (chx04151), “NF-kappa B signaling pathway” (chx04064), and “p53 signaling pathway” (chx04115) (Figure 4E). Through KEGG enrichment analysis and the manual curation of DEGs, we determined that the primary alterations in heat-stress-treated SCs are predominantly concentrated in metabolic processes and immune–inflammatory responses.

**Figure 5:**
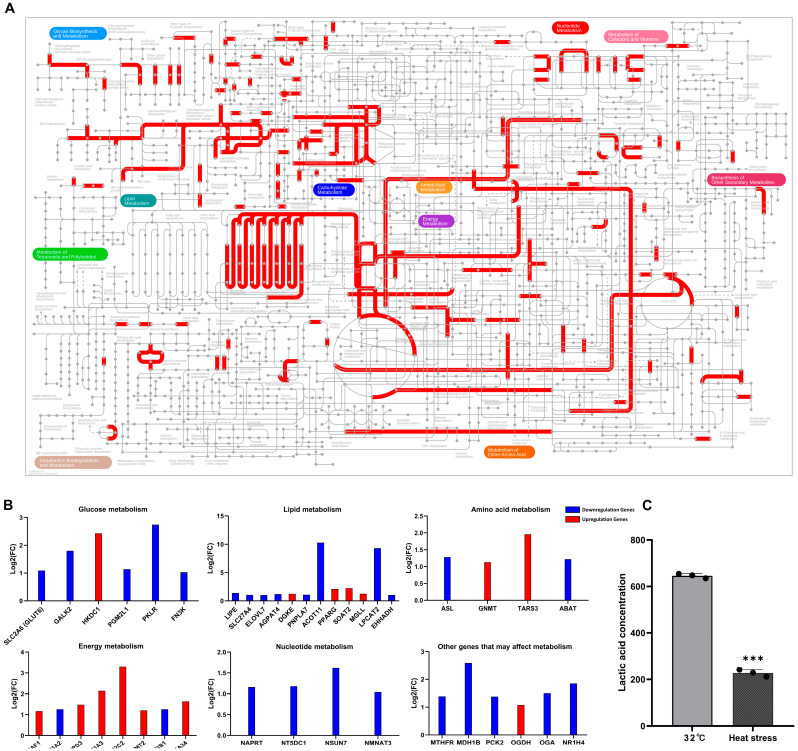
Heat stress activates multiple metabolic pathways in goat testicular SCs and affects lactate production. (**A**) A metabolic pathway map of DEGs based on iPath3 website analysis. Red lines indicate metabolic pathways activated by heat stress. (**B**) A bar graph of DEGs related to various metabolic processes in the heat-stress group. Shows expression changes in genes associated with glucose metabolism, lipid metabolism, amino acid metabolism, energy metabolism, and nucleotide metabolism. (**C**) The effect of heat stress on lactate content in SCs’ culture supernatant. The bar graph compares lactate concentrations between the control and heat-stress groups. *** *p* < 0.001.

Metabolic pathway-related DEGs were analyzed using the iPATH3 website (http://pathways.embl.de, accessed on 30 June 2024). Transcriptomic analysis revealed extensive metabolic reprogramming in heat-stressed SCs (Figure 5A). KEGG pathway analysis highlighted significant changes in glucose, lipid, amino acid, energy, and nucleotide metabolism (Figure 5B). We observed the downregulation of *PKLR* in glucose metabolism and *ACOT11* and *LPCT12* in lipid metabolism (|Log2(FC)| > 2, *p* < 0.05). Conversely, ATP1A3 and ATP1C2, involved in energy metabolism, were upregulated. Genes related to nucleotide metabolism and other pathways showed varying degrees of downregulation. Given SCs’ crucial role in lactate production for spermatogenesis, we measured lactate levels (Figure 5C) and found a significant decrease in secretion following heat stress (*p* < 0.05).

A transcriptomic analysis of heat-stressed SCs revealed a multifaceted stress response (Table 1). Cell cycle regulation was altered, with *RPRM* upregulation (Log2FC = 2.54) and *GADD45G*, as well as *TP53I3* downregulation (Log2FC < −1), suggesting cell cycle arrest. Notably, a strong inflammatory and immune response was observed, with a substantial upregulation of chemokines (*CXCL10*, Log2FC = 4.91; *CCL20*, Log2FC = 3.55; *CCL2*, Log2FC = 2.66), cytokines (*LTA*, Log2FC = 4.09), and inflammatory mediators (*PTGS2*, Log2FC = 2.09; *MMP3*, Log2FC = 3.23). The anti-apoptotic gene *BCL2* was downregulated (Log2FC = −1.42), while *PIK3CD*, involved in cell survival pathways, showed slight downregulation (Log2FC = −1.05). These changes collectively indicate a pronounced inflammatory state coupled with altered cell survival mechanisms, potentially impacting SCs’ function and spermatogenesis.

### 3.5. PPI Network of DEGs Under Heat Stress

**Figure 6:**
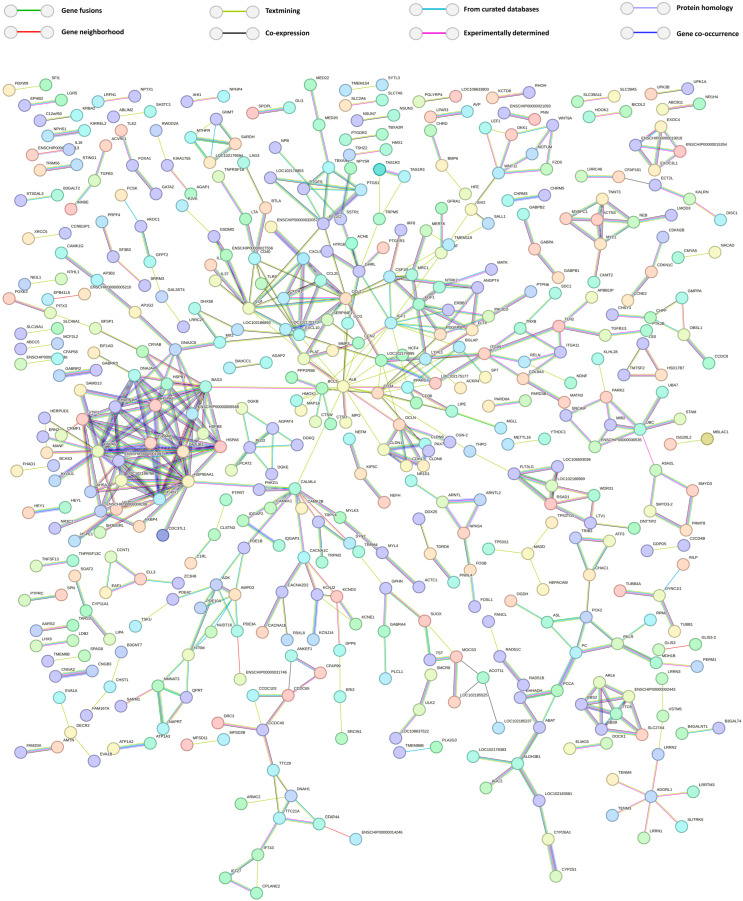
STRING interaction analysis of all differentially expressed genes DEGs between 32 °C and heat-stressed SCs. Protein–protein interaction network visualized using STRING, with interactions shown at a confidence level of 0.4, while edges between nodes indicate various types of interactions, color-coded and defined in the figure legend. Isolated nodes without edges were removed from the visualization.

Subsequently, we performed a functional protein–protein interaction network analysis for the proteins encoded by the DEGs. The network was constructed with medium confidence (0.4) and visualized in Figure 6. It comprised 1144 nodes with 224 interactions (edges). The average local clustering coefficient of the network was 0.149, and the PPI enrichment *p*-value was less than 1.0 × 10^−16^, indicating a highly significant level of interaction among the differentially expressed proteins.

**Figure 7:**
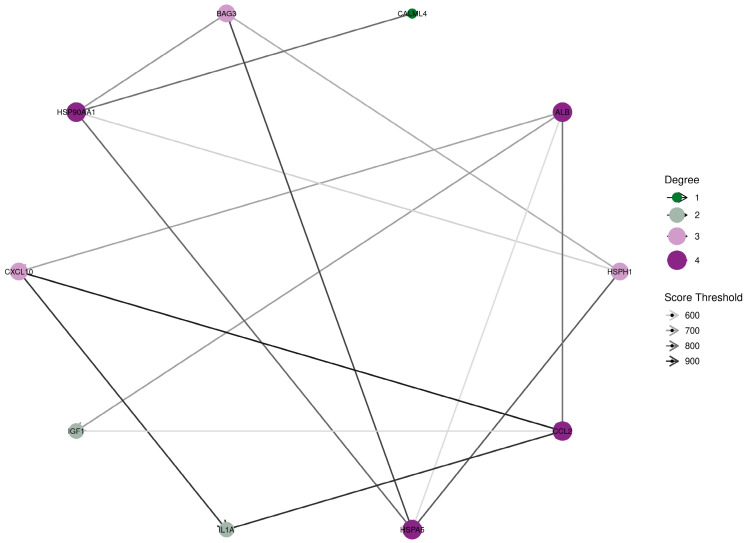
Core genes related to heat-stress injury associated with SCs. Node scores were calculated using Cytoscape software, and the top 10 nodes ranked by node scores were selected.

To identify the key players in this network, we calculated node scores using Cytoscape software(Version 3.10.2) and selected the top 10 nodes based on their scores (Figure 7). The identified Top 10 hub genes were *HSP90AA1*, *HSPA5*, *BAG3*, *IGF1*, *HSPH1*, *IL1A*, *CCL2*, *CXCL10*, *ALB*, and *CALML4*. These hub genes likely play crucial roles in the heat-stress response of SCs and may serve as potential targets for further investigation.

## 4. Discussion

This study established an in vitro heat-stress model to thoroughly investigate the effects of heat stress on goat testicular SCs and its potential mechanisms. Our results reveal significant physiological and molecular changes in SCs induced by heat stress, including decreased cell viability, elevated ROS levels, increased autophagy, and extensive gene expression alterations.

The transcriptome analysis identified 1231 DEGs involved in several key pathways, such as the PI3K-Akt signaling pathway, NF-kappa B signaling pathway, and p53 signaling pathway. The activation of these pathways suggests that heat stress may affect SC function through multiple mechanisms. Notably, we observed significant changes in the expression of genes related to inflammation, apoptosis, cytoskeleton, and cell adhesion, which may explain the functional impairment of SCs caused by heat stress. The combined effect of these pathways on Sertoli cell function could explain the observed decrease in cell viability, increased ROS levels, and altered gene expression patterns. Moreover, this intricate signaling network may influence the cells’ ability to support spermatogenesis by affecting their metabolic functions, secretory activities, and structural integrity. Understanding these pathway interactions provides a more comprehensive view of how heat stress impairs Sertoli cell function and opens up potential avenues for targeted interventions to mitigate heat-stress effects.

Furthermore, our study provides a detailed description of heat-stress-induced metabolic reprogramming in goat SCs. We observed changes in gene expression related to glucose metabolism, lipid metabolism, and energy metabolism, with a significant decrease in lactate secretion in particular, which may directly affect spermatogenesis. This finding offers new insights into understanding how heat stress impacts the ability of SCs to support spermatogenesis. These metabolic alterations not only explain the reduced lactate secretion but also highlight a broader impairment of Sertoli cell function under heat stress. The resulting nutrient and energy deprivation in the seminiferous epithelium likely contributes significantly to the observed decline in spermatogenesis efficiency under heat-stress conditions.

Heat stress induces various inflammatory responses in animals, and a complex interplay exists between inflammation and autophagy pathways [21,22]. Inflammation induced by heat stress can suppress cellular autophagy levels, thereby amplifying the inflammatory response. Conversely, autophagy participates in the negative feedback regulation of inflammatory responses by degrading inflammatory factors and modulators [23,24]. This bidirectional regulatory mechanism reveals the delicate balance maintained by organisms when coping with heat stress [25,26,27]. Notably, excessive inflammation can lead to organ and tissue damage, potentially resulting in inflammatory diseases [28]. In such cases, enhancing autophagy levels can effectively alleviate inflammation-induced damage [29,30,31]. This mechanism provides important insights for understanding heat-stress response strategies and developing potential therapeutic approaches.

PPI network analysis further identified 10 key hub genes, namely *HSP90AA1*, *HSPA5*, *BAG3*, *IGF1*, *HSPH1*, *IL1A*, *CCL2*, *CXCL10*, *ALB*, and *CALML4*. When cells encounter heat stress, these genes play pivotal roles in diverse biological processes, including heat-shock response, cellular protection, and inflammatory reactions. *HSP90AA1* encodes heat-shock protein 90α, a molecular chaperone that aids in the correct folding of other proteins, thereby maintaining intracellular protein stability [32]. *HSPA5*, also known as *GRP78*, is an endoplasmic reticulum heat-shock protein involved in protein folding and cellular stress responses [33,34,35]. *BAG3* is an anti-apoptotic protein that interacts with heat-shock proteins to help cells cope with heat stress [36]. *IGF1*, or insulin-like growth factor 1, plays a crucial role in regulating cell growth, survival, and metabolism [37]. *HSPH1* (also called HSP105) is involved in protein folding and cellular protection [38,39], while *IL1A* acts as a pro-inflammatory cytokine involved in the inflammatory response induced by heat stress. Both *CCL2* and *CXCL10* are chemokines that play roles in inflammatory responses and immune cell chemotaxis, respectively [40]. *ALB* encodes albumin, the main protein in plasma, which may have a protective role during heat stress [41]. *CALML4*, as calmodulin-like protein 4, may be involved in calcium signaling, although its specific role in heat stress requires further study [42,43]. These genes work synergistically to help cells manage heat stress, protect cellular components, and regulate stress and inflammatory processes, with their expression changes reflecting the adaptive response of cells to heat stress. These genes likely play crucial roles in the heat-stress response, providing important targets for further research and potential intervention strategies. These hub genes likely work in concert to orchestrate a comprehensive cellular response to heat stress. For instance, the heat-shock proteins (*HSP90AA1*, *HSPA5*, *HSPH1*) may collaborate to maintain protein homeostasis, while BAG3 could modulate their activity and promote cell survival. Simultaneously, the inflammatory mediators (*IL1A*, *CCL2*, *CXCL10*) might interact with IGF1 to balance cell protection and stress signaling, collectively enabling Sertoli cells to adapt to and mitigate the detrimental effects of heat stress.

In conclusion, this study not only deepens our understanding of how heat stress affects SC function but also provides new insights for developing protective strategies. Enhancing autophagy levels may be an effective approach to alleviate inflammation-induced damage caused by heat stress. However, given the complexity of the heat-stress response, future research needs to further explore the specific regulatory mechanisms of these pathways and how these findings can be translated into practical applications in animal husbandry to address the challenges posed by global climate change.

## 5. Conclusions

Our study reveals that acute heat stress in vitro significantly impairs SCs function, inducing reduced viability, increased oxidative stress, and enhanced autophagy. The observed interplay between inflammatory signaling and cell cycle pathways, along with suppressed transcriptional and proliferative activity, underscores the critical role of inflammation–metabolism interactions in determining the SCs’ fate under heat stress. These findings highlight the importance of reproductive management in heat-stress conditions.

## Figures and Tables

**Table 1:** The top 15 genes related to immune inflammation and cell apoptosis in transcriptome DEGs.

Genes	Full Name	LogFC	Description
*PTGS2*	Prostaglandin-Endoperoxide Synthase 2	2.087813348	Inflammatory mediator production, involved in inflammatory response
*CCL2*	C-C Motif Chemokine Ligand 2	2.655211633	Chemokine, attracts monocytes and T cells
*MMP3*	Matrix Metallopeptidase 3	3.230963759	Extracellular matrix remodeling, involved in tissue restructuring and inflammation
*CXCL10*	C-X-C Motif Chemokine Ligand 10	4.908751369	Chemokine, attracts T cells and NK cells
*LTA*	Lymphotoxin Alpha	4.088700229	Cytokine, regulates immune response and inflammation
*CCL20*	C-C Motif Chemokine Ligand 20	3.54863778	Chemokine, attracts dendritic cells and T cells
*LAT*	Linker For Activation of T Cells	3.526152059	T cell activation signal transduction
*RRM2*	Ribonucleotide Reductase Regulatory Subunit M2	−1.011569308	Involved in DNA synthesis and repair
*SERPINE1*	Serpin Family E Member 1	1.516341237	Regulates blood coagulation and cell adhesion, involved in tissue remodeling
*GADD45G*	Growth Arrest And DNA Damage Inducible Gamma	−1.064511721	Cell cycle arrest and DNA damage response
*TP5313*	Tumor Protein P53 Inducible Protein 3	−1.216863438	p53 pathway, involved in cell cycle regulation and apoptosis
*RPRM*	Reprimo, TP53 Dependent G2 Arrest Mediator	2.536588167	p53-dependent cell cycle arrest
*BCL2*	BCL2 Apoptosis Regulator	−1.41514299	Anti-apoptotic protein, regulates cell survival
*IGF1*	Insulin Like Growth Factor 1	−1.035502139	Growth factor, promotes cell growth and survival
*PIK3CD*	Phosphatidylinositol-4,5-Bisphosphate 3-Kinase Catalytic Subunit Delta	−1.049545573	Involved in cell survival, proliferation, and differentiation signaling pathways

## Data Availability

The authors declare that the data supporting the findings of this study are available within the paper.

## Supplementary Materials

The following supporting information can be downloaded at https://www.mdpi.com/article/10.3390/genes15121582/s1: Table S1. List of primers required for RT qPCR. Table S2. Significant transcriptome differentially expressed genes.
