# Supplementary material for: Transcriptome Analysis Reveals Sertoli Cells Adapting Through Redox and Metabolic Pathways Under Heat Stress in Goats
Source: Genes (Basel). 2024 Dec 9;15(12):1582. doi: 10.3390/genes15121582 (PMC11675638; doi:10.3390/genes15121582)
Supplement: Supplementary file 1 [file genes-15-01582-s001.zip › Supplementary Materials 1.pdf]

Table S1. List of Primers Required for RT qPCR.

Gene Name	Direction	Sequence
PKD1	FORWARD	GCAGGACTACGAGATGGTGGAG
	REVERSE	AAGCGGACTTTGTGGCGAAAC
HSPB8	FORWARD	CGCCCAGCCCACGAGAAG
	REVERSE	CCGCCACTGTCCTCCCAAG
RGS4	FORWARD	TGGGTCAGAGAAGCAGAAAGGAG
	REVERSE	CAGGCATAGTCTTGGCATCTCAG
CRYBG1	FORWARD	GGGAGCATACCGCCGAAGG
	REVERSE	CGAAGCCGAAGAGTCCTCAGG
DSEL	FORWARD	TCTGTGCCTGCCCTGTGAAG
	REVERSE	GCCTCTCCTCGGTGGTAATCG
GATD1	FORWARD	CCCGCAGACCCGCCATG
	REVERSE	GCGTGAAGCAGTGCAGGAAG
HSPH1	FORWARD	GTGAGCAAGCAGCGGAAGATC
	REVERSE	TGGTGGTTCGCAGAGCAGTAG
CCN2	FORWARD	TCACAGAACCACCACCCTTCC
	REVERSE	GTAATGGCAGGCACAGGTCTTG
GREB1	FORWARD	GCCTGCCTGACCATCTCCTG
	REVERSE	GCCACAGCCGACACAATTCC
MANF	FORWARD	CCGTGAAGCAAGAGGCAAAGAG
	REVERSE	GGCTTGGACACCTCGTTGATG
