# Supplementary material for: Transcriptome Analysis Reveals Sertoli Cells Adapting Through Redox and Metabolic Pathways Under Heat Stress in Goats
Source: Genes (Basel). 2024 Dec 9;15(12):1582. doi: 10.3390/genes15121582 (PMC11675638; doi:10.3390/genes15121582)
Supplement: Supplementary file 1 [file genes-15-01582-s001.zip › Supplementary Materials 2.pdf]

Table S2. Significant transcriptome differentially expressed genes.

gene_name	log2(fc)
AHSA1	1.57
HSPA5	2.02
BAG3	2.96
DNAJA4	4.88
FKBP4	2.16
CRYAB	3.71
HSPB1	3.39
CCN2	-1.56
HSPB8	7.10
SERPINH1	1.95
CACYBP	2.67
HSPH1	4.00
ENSCHIG00000023424	2.61
ENSCHIG00000019796	5.57
HSP90AA1	1.94
HSP70.1	5.72
ENSCHIG00000018001	3.15
HSPA8	1.50
KIAA2013	1.83
ENSCHIG00000004382	1.33
HSPA6	7.50
UPK3B	-2.31
HMOX1	1.43
INHBE	-4.55
HSPA4L	2.68
ENSCHIG00000022054	1.67
EAF1	1.07
YTHDC1	1.45
CLDN1	-1.17
DSEL	1.81
CRYBG1	1.66
ABCC5	1.76
PDLIM3	-1.15
ENSCHIG00000009467	1.33
GMEB2	1.51
ENSCHIG00000010802	1.14
P4HA1	1.09
ENSCHIG00000017456	3.27
ENSCHIG00000010216	1.05
UBTF	1.10

GFPT2	-1.06
MIDN	1.10
PTPN6	3.91
ASH2L	1.14
CHPF	-1.52
PTGS2	2.09
RAE1	1.27
PCK2	-1.38
MANF	1.05
NDRG1	1.12
OGA	1.50
HERPUD1	-1.13
MVP	-1.23
SF3B3	-1.06
TGFB1I1	-1.99
CEMIP	-1.69
GABPB1	1.93
HYOU1	1.09
GPHN	-1.74
DOCK1	-1.33
DEPDC7	1.89
TBC1D5	-2.42
ENSCHIG00000024648	1.26
PNN	1.00
CCL2	-2.87
BAHCC1	1.28
OGDH	-1.08
DNTTIP2	1.02
LTV1	1.19
AKAP8L	1.26
TGFB3	-1.33
BAIAP2	1.16
CD248	-1.22
IL1RL1	1.56
RNF187	-2.00
ISLR2	-1.61
IRF8	2.96
CHAC1	-2.68
METRNL	1.28
PDGFRB	-1.28
GREB1	-1.47
LTO1	1.15
CRISPLD2	-1.38

FAM114A1	-1.24
ARAP1	-1.17
GPRC5A	1.46
VCAN	-1.09
ATP10A	-1.42
ISG20L2	1.01
LEF1	1.14
RBBP6	1.05
PKD1	-1.10
SH3BGR	4.31
NR4A3	3.24
MAP1A	-1.08
HACL1	-1.50
SLC25A22	1.06
EDRF1	1.21
PIH1D2	2.01
PCCA	-1.29
ITPRIP	1.23
RGS4	-1.13
OBSL1	-1.19
CAMKK1	1.16
NLRX1	-1.07
RCOR2	1.53
CC2D1B	-1.09
MOV10	-1.00
BBS2	-1.42
TTLL5	-1.27
GATD1	-1.58
ARHGAP31	1.06
PC	-1.34
H2AC6	3.21
MFSD11	1.04
PDLIM2	-1.19
ENSCHIG00000007799	-1.43
SELENOO	-1.29
ENSCHIG00000022585	-1.21
BANP	1.26
GABPA	1.23
TSPAN18	-1.88
NAGA	-1.06
LMBRD1	-1.00
NASP	-1.12
ENSCHIG00000021699	-1.10

NUFIP2	1.17
ESYT2	-1.00
DACT3	-1.75
SUOX	-1.29
CHORDC1	1.10
NPRL3	-1.65
ADAMTS14	-1.56
OSBPL7	1.23
WDR37	1.13
COBLL1	1.23
ENSCHIG00000022343	1.47
HEY1	2.39
EVA1B	-1.14
ICA1	-1.21
ENSCHIG00000015125	1.32
MAP2K5	-1.22
GSDMD	-1.35
UAP1L1	-1.09
MADD	-1.31
CBX6	1.16
TMEM119	-1.25
ATP1A2	-1.26
KIAA0319L	-1.15
CD34	-1.53
TNFRSF1B	1.15
ZNF292	1.43
CPXM1	-1.35
SOGA1	1.48
FOSB	2.12
ENSCHIG00000000015	1.10
C4orf54	1.44
ADAMTS7	-1.14
PEAR1	-1.34
NCKAP5	-2.40
MANSC1	-1.21
FOSL1	1.10
ENSCHIG00000016367	-1.36
L3MBTL3	1.31
ADK	-1.05
KRCC1	-1.02
STAM	1.06
TSKU	-1.28
ZNHIT6	1.02

ENSCHIG00000024010	-1.10
ENSCHIG00000009551	2.17
OTULINL	-1.62
PLAT	-1.02
EPHB2	-1.08
NHLRC2	1.16
PPP6R2	-1.00
SLC6A6	-1.20
B3GALT4	-1.31
ENSCHIG00000016194	-2.53
GMPPA	-1.03
CNPY4	-1.36
CCDC137	1.07
BCAS3	-1.44
ERI3	-1.06
MYCN	-1.28
HOMER3	-1.21
STYXL2	-1.55
C15orf39	1.06
ENSCHIG00000021602	1.18
GMEB1	1.13
POLD2	-1.06
TRAPPC9	-1.31
BEND3	1.57
ENSCHIG00000015655	-1.03
PARD3B	-1.08
CDC37L1	1.04
EHD3	-1.27
PTGS1	-1.62
GDPD5	-1.16
ENSCHIG00000019793	-1.84
MIB2	-1.16
CYP11A1	-1.15
CMTM3	-1.42
ENSCHIG00000026059	-1.38
ENSCHIG00000010681	1.07
LIPA	-1.13
XRCC5	-1.03
AHI1	-1.29
IL1A	1.68
GSTZ1	-1.46
ENSCHIG00000021886	-1.65
KLHDC9	-1.55

KATNIP	-1.80
ENSCHIG00000016489	1.03
DHX58	-1.06
TRPM4	-1.54
PPP1R16A	-1.17
SEMA3C	-1.42
LSS	-1.04
ENSCHIG00000010967	2.31
AP1G2	-1.01
PRPSAP2	-1.11
PRDM15	1.18
IQGAP3	-1.29
CDK10	-1.26
MOCS3	1.01
ROBO3	-1.11
LPAR3	-1.02
USPL1	1.14
MED22	1.17
GALK2	-1.80
ANTXR1	-1.07
DNAJC22	-1.34
SDC1	-1.05
ALG8	-1.23
METTL1	-1.35
ENSCHIG00000009248	3.67
PEX7	-1.06
ENSCHIG00000013777	-1.59
LCOR	1.37
AMIGO3	1.57
KIF5C	-1.22
CGNL1	-1.25
PDE10A	1.27
TPGS1	-1.00
KLHL28	1.66
EIF1AD	1.04
VGLL2	-1.14
NAV3	1.02
TST	-1.23
NUDT16	-1.37
ZBTB11	1.08
ZNF304	1.03
ENSCHIG00000026521	1.40
DYNC2I2	-1.11

PRDM12	2.05
RPS6KA2	-1.01
TTC8	-1.71
SLC7A6	1.14
ZC3H8	1.12
RALGPS2	1.51
CNGA3	-1.25
TRIM56	1.17
GJA5	-1.24
THSD4	-1.59
RRM2	-1.01
GLCE	-1.13
TBCK	-1.20
ST3GAL3	-1.02
DAAM2	-1.69
ENOX1	-1.76
ZNF84	-1.65
BRSK1	-1.67
SMCR8	1.04
PKNOX2	-1.81
NT5M	-1.23
PDE3A	1.45
RFTN1	-1.23
BTLA	3.31
TANGO6	-1.07
UBA7	-1.08
SLITRK5	2.20
WFIKKN2	-1.31
ENSCHIG00000019468	-1.31
ENSCHIG00000001017	2.55
MINAR1	3.31
LHPP	-1.05
QPRT	-1.16
TCF21	-1.01
PLD2	-1.11
ENSCHIG00000023433	-1.18
SLC37A4	-1.21
SARDH	-1.07
MATN3	3.45
UBC	-1.24
ESM1	1.06
ENSCHIG00000027195	1.08
CCDC107	-1.34

RCSD1	-1.07
AGAP1	-1.18
IFT27	-1.04
TKFC	-1.10
ADAT2	1.07
AARS2	-1.34
SERPINE1	1.52
CXCL5	-1.17
FBXO4	-1.79
RNF135	-1.10
TMEM33	1.19
NPY5R	1.45
LRRN2	-1.34
RFFL	-1.29
MED20	1.09
POLR1G	1.46
CHRD	1.26
NTHL1	-1.10
ATF3	1.08
ENSCHIG00000011999	1.11
SHFL	-1.18
ENSCHIG00000018200	-1.18
ENSCHIG00000015230	1.03
LGR5	-1.19
ENSCHIG00000024286	1.44
KALRN	-1.30
CCDC136	-1.98
SETDB2	1.11
SLC19A1	-1.04
RALGAPA2	-1.16
TRIB3	-1.25
CACNA1C	-1.32
SLC8B1	-1.39
MTF1	1.05
TRERF1	1.19
GGACT	-1.09
GASK1A	-2.31
POLM	-1.03
ENSCHIG00000021095	-1.01
ZCCHC2	1.28
CDH3	-1.04
ALPK1	-1.25
ICOSLG	-1.49

ADAMTS17	-1.43
IPPK	1.03
CNTLN	-1.51
CDKN1C	-1.25
GLB1L	-1.12
ENKD1	-1.25
PARP9	-1.06
STARD10	-1.19
ADAM12	-1.03
NAA80	-1.09
PGGHG	-1.30
TENT5C	1.84
KLHL14	1.85
ADGRD1	-1.89
ENSCHIG00000017153	1.18
MTSS1	1.03
ADGRL1	-1.15
GEMIN6	-1.16
CACFD1	-1.25
CCNT1	1.21
CCDC8	-1.21
WDR54	-1.35
MPZL3	1.09
B3GNT9	-1.43
ADAMTS9	-1.19
ENSCHIG00000018071	1.15
TBC1D4	-1.38
ENSCHIG00000013541	1.03
OLFML1	-1.57
PERM1	4.46
EXTL2	-1.15
SMYD3	-1.73
SNORA73	1.51
YPEL2	-1.54
OSR1	1.17
RSAD1	-1.48
ENSCHIG00000010424	-1.06
EXOC3L1	-1.19
GAL3ST4	-1.17
ULK2	-1.01
TUBB4A	-1.62
SLC39A11	-1.06
MMP3	12.96

PTGIR	-1.96
SCAPER	-1.34
NAPRT	-1.16
IGDCC4	-1.89
ARNTL	-1.18
FAM78A	-1.98
TLN2	-1.04
ISLR	-1.08
ENSCHIG00000020381	-1.31
ENSCHIG00000008044	1.59
RWDD2A	-1.37
H1-6	3.06
SYPL2	-1.18
FAM184A	-1.10
FBXW9	-1.18
LIPE	-1.38
KRBA2	-1.74
HSPA1L	1.22
GADD45G	-1.06
AUH	-1.03
TM7SF2	-1.33
TECPR1	-1.26
NPFFR2	2.28
TMEM266	-1.14
ENSCHIG00000008571	-2.79
VASH1	-1.31
CCDC190	-1.98
ZNF654	1.02
TENM3	-1.11
CCNB1IP1	1.55
TNFAIP8	1.20
DUSP23	-1.45
SLC16A10	1.78
ENSCHIG00000026843	-1.04
CDKN2B	-1.10
SLC27A4	-1.04
TP53I3	-1.22
NR1D1	1.18
MX2	-1.18
LHX9	1.56
ARHGEF25	-1.02
C1QTNF6	-1.04
ENSCHIG00000006848	1.10

ENSCHIG00000022263	1.52
PPP2R5B	-1.12
AGAP2	-2.88
MTHFR	-1.39
FBXL4	-1.20
ENSCHIG00000020956	1.13
BMP6	-1.23
SIX2	1.28
ENSCHIG00000012177	1.16
FILIP1	-1.51
HPGD	2.95
FLYWCH1	-1.04
MXRA8	-1.10
SDHAF1	1.17
GJB3	1.10
ENSCHIG00000019902	-1.10
SNX29	-1.91
ENSCHIG00000011276	1.25
TSPAN11	-1.26
SNORD22	2.16
ULK4	-1.06
ENSCHIG00000017158	1.48
ENSCHIG00000009645	1.42
USP37	1.26
TMEM102	-1.92
MBLAC1	-1.07
REM1	-1.63
ENSCHIG00000001686	-1.90
KLHL42	-1.07
TLE2	-1.23
ENSCHIG00000017031	1.73
SPIRE2	-1.11
FCSK	-1.06
CTC1	-1.05
PTGES	-1.26
HSD17B7	-1.41
EMP2	-1.02
RNF34	-1.16
ENSCHIG00000013918	-1.21
HOOK2	-1.17
NEFM	1.46
CASKIN1	2.62
LIMD2	-1.22

SDCCAG8	-1.49
ASL	-1.28
NAIF1	1.12
HFE	-1.03
TMED6	1.10
DGKQ	-1.30
ITGA11	-1.57
ENSCHIG00000009337	-2.05
SEC22C	-1.61
SPOPL	1.00
NPHP4	-1.12
CCDC81	4.04
LRRN1	-1.33
TPH2	1.37
7SK	1.99
ENSCHIG00000005750	-2.16
SOX11	1.15
GALNT12	-1.29
ENSCHIG00000013624	-1.03
VSTM4	-1.42
SCRN2	-1.05
ENSCHIG00000018109	-1.83
TMEM200A	1.15
ENSCHIG00000023778	-1.30
ARHGAP27	2.01
AKAP6	1.65
CCDC157	-1.79
CFAP161	5.42
NBEA	-1.14
IFT43	-1.33
PLCL1	1.12
SCARA5	-1.18
CD40	-1.30
KRT80	-1.42
FLT3LG	-1.36
TMEM106A	-1.38
ACHE	1.42
RND1	-1.72
COL11A2	1.88
NREP	-1.10
ELMO3	-1.09
LRRTM3	1.67
CABCOCO1	-1.08

PGBD2	1.11
FOXF2	3.78
SH2D3C	-1.64
MYLK3	1.19
AADACL3	-1.37
PLEKHA6	-1.47
DAPK2	-1.28
SLC2A6	-1.09
ENSCHIG00000019787	-1.14
CTSO	-1.14
ADAMTSL4	-1.15
CDH1	-1.10
AGPAT4	-1.20
MYBPH	-1.13
FAM126B	1.04
NCKAP1L	-2.25
ENSCHIG00000016081	-1.30
HSD11B2	-1.26
ENSCHIG00000026867	-1.25
PROX2	-1.93
TBC1D32	-1.01
ARHGAP44	-1.33
ELOVL7	-1.02
CHRM3	1.10
NOTUM	2.71
CCNE2	-1.10
CRMP1	-2.51
SNORD22	1.05
SPRN	-2.22
SSH2	1.05
ENSCHIG00000009600	11.74
ATP8B4	-2.03
ZDHHC14	-1.07
MEDAG	1.93
DPH3	1.06
ENSCHIG00000021044	-2.20
ENSCHIG00000018980	-1.03
AOC2	1.18
FLYWCH2	-1.11
MYL1	-13.10
SOCS2	-1.27
ARRDC5	12.51
OCLN	-1.29

VEPH1	-1.01
NPPB	-12.88
SNORA73	1.76
MEIS3	-1.45
ENSCHIG00000023160	-1.15
PTGER3	2.39
TDRD6	2.43
MAP7	-1.16
JAKMIP2	-2.21
SOX8	1.35
ENSCHIG00000022883	1.09
MYCBPAP	-1.27
DECR2	-1.01
DND1	1.21
PARP6	1.15
ENSCHIG00000026755	1.53
RAD51C	-1.10
ENSCHIG00000011637	-1.02
ZFP1	-1.05
HTR1B	1.79
NEB	1.01
TMEM235	-1.27
TRPV4	-1.90
MYCL	2.50
KCNK1	-1.45
FAIM2	3.36
GABPB2	1.44
NDNF	1.34
MAMDC2	-1.07
PCDH10	1.86
C3orf14	-1.07
EPM2A	-1.23
GNAL	11.89
XRRA1	-1.21
MYORG	-1.46
ENSCHIG00000021894	1.54
HNMT	-1.13
RPRM	2.54
ZNF784	-1.18
ENSCHIG00000013760	-1.15
C1RL	-1.26
TRPM5	10.17
SARM1	-1.92

AMPD3	1.48
SNORD54	1.82
TRIM52	1.22
SLC16A3	-1.43
ENSCHIG00000024343	1.11
F2RL1	-1.09
MEP1B	10.56
ENSCHIG00000013428	-1.35
RGCC	-1.46
NT5DC1	-1.18
PPCDC	-1.10
NSUN7	-1.62
PIWIL4	10.62
NPHS1	-1.12
SH3TC1	1.08
XKR5	-1.17
MELTF	-1.16
ZNF565	1.12
HES7	2.03
ENSCHIG00000011310	1.49
ENSCHIG00000018690	-1.48
CPLANE2	-1.07
KLF9	-1.58
ENSCHIG00000005803	1.02
GDNF	1.78
CBY2	-10.37
ZNF853	-1.90
MAN1C1	-1.08
ENSCHIG00000013167	-1.40
ZBTB6	1.05
ENSCHIG00000027018	-2.42
NSUN3	-1.13
RIPOR3	1.44
MYH14	1.35
ENSCHIG00000007607	2.92
MCAM	-2.35
CD36	2.02
TSSK4	2.24
PPFIA3	-1.36
PADI1	1.91
CMYA5	1.21
ENSCHIG00000015360	1.66
NRCAM	1.19

TREM1	-1.59
ENSCHIG00000021047	1.83
TTC21A	-1.06
HEYL	-1.27
TAS1R2	10.44
TRIM14	-1.16
ENSCHIG00000021073	-1.23
FAIM	-1.07
GJC2	2.42
PCDHGA4	-1.10
TUBB1	5.00
TP53I11	-1.70
ZNF536	1.27
FSIP1	-10.71
TNK1	-1.47
NEIL1	-1.58
OSR2	1.24
ENSCHIG00000023691	1.19
SLC25A35	-1.31
SHPK	-1.50
VMAC	-1.79
SP7	-9.78
CTF1	-1.43
ENSCHIG00000007164	-1.31
CPM	1.94
GCNT7	1.89
NKX6-2	11.33
EDNRB	1.76
ABCA6	-1.16
CHST1	-1.41
ENSCHIG00000019534	1.03
ACTN3	-1.45
DNAH12	4.17
CXCL10	-11.26
FBXL2	-1.16
DPP6	10.16
ENSCHIG00000016905	13.58
ABCB11	8.73
ENSCHIG00000024836	2.15
EYA4	1.61
FRRS1	-1.26
SEC14L4	-11.45
MDH1B	-2.59

HPN	11.15
BEST2	10.94
ENSCHIG00000015139	-1.17
ENSCHIG00000022644	3.16
SRRM3	2.74
DGKB	-1.92
CORO2B	-1.50
ENSCHIG00000026103	8.99
HMX1	4.06
PRR35	10.78
UBN2	1.06
KLF2	-1.50
ENSCHIG00000008026	-11.97
SNORD31	1.67
ZBTB43	1.02
ENSCHIG00000014138	-1.01
THPO	1.65
GABRR2	3.26
UPK1A	11.86
SNORD12B	1.16
LMOD3	4.16
GPR35	-11.79
ASXL3	1.12
ENSCHIG00000024590	-11.96
CHRM5	6.97
SV2C	10.18
ENSCHIG00000009869	-1.14
IQCH	-1.59
FOXC1	1.45
CARMIL3	-9.58
DDX25	-1.63
WDR31	-1.56
ENSCHIG00000006991	-1.09
ENSCHIG00000018052	-2.03
KIAA1755	-2.40
ENSCHIG00000002993	1.29
KCNK7	1.84
IQGAP2	1.47
ENSCHIG00000012101	-1.39
MCF2L2	1.13
DGKE	1.23
MDGA1	1.34
H2BU1	2.39

NWD2	1.13
ENSCHIG00000014514	-1.95
SNORA61	1.02
CTCFL	9.10
PRSS33	-11.74
C10orf143	-1.01
ENSCHIG00000005311	-1.32
ENSCHIG00000009496	1.08
CD101	1.43
CYP26A1	1.62
PIGH	-1.29
KIRREL2	-3.16
HCRTR1	-1.18
IFITM10	3.06
LRRC46	-1.16
CDCA7	-1.09
PGM2L1	-1.14
BHLHE22	-2.33
ACTC1	-1.04
PLS1	1.45
DNAH1	1.96
SLC7A11	1.25
CCDC103	-2.06
ENSCHIG00000006533	1.25
ENSCHIG00000007867	9.78
ENSCHIG00000015243	1.85
SLC46A1	-1.01
CEP85L	1.16
CALML4	-1.22
SMPDL3B	-1.89
SCARNA12	1.64
ENSCHIG00000022140	1.25
ENSCHIG00000008310	3.77
DLX4	1.38
SCN7A	4.67
SPTBN5	1.96
HEPACAM	-1.29
PRMT8	-2.95
DRC3	-10.73
BFSP1	-3.36
MPP2	-2.56
CLSTN3	-1.15
TLX2	-2.40

NOSTRIN	-1.25
GLYATL3	3.22
ENSCHIG00000001247	2.25
FBXL8	-1.37
KCNK3	-1.28
RAB3IL1	-1.03
MYZAP	-1.15
HLA-DMA	1.20
ATP2C2	3.30
CSF2RB	-3.11
SNORD65	1.62
ENSCHIG00000007993	8.80
ARNTL2	-1.04
PLEKHG6	1.61
TLR9	2.53
MFSD2B	1.64
ENSCHIG00000014424	4.21
TBXA2R	-1.37
TP53TG5	1.96
PCDHGA6	-1.06
NMB	-12.68
ENSCHIG00000009834	1.00
ENSCHIG00000014814	-10.17
DUOXA1	-1.50
SSTR1	1.77
ENSCHIG00000012058	11.95
ENSCHIG00000008226	9.46
SMOC1	-1.17
ENSCHIG00000011109	4.52
HMCN2	3.96
ENSCHIG00000017993	1.12
MYL4	2.00
MAB21L2	1.54
ENSCHIG00000026175	-1.60
PLA2G3	-1.88
LMAN1L	3.49
ENSCHIG00000016416	-2.44
TNNT3	3.17
CFAP99	3.24
MOB3B	1.37
PNPLA7	-1.08
LYVE1	1.80
MYO15A	3.21

ENSCHIG00000023338	1.55
RGS6	-1.15
ENSCHIG00000025260	-10.39
SPEF1	1.41
CUX2	-2.00
CORO6	1.03
BATF2	-2.01
RAX2	11.86
ACOT11	-10.29
BCL2	-1.42
CRNN	10.18
CKMT2	1.21
TMEM52	-1.13
RILP	-1.04
ENSCHIG00000001183	-1.03
DNAJC6	1.50
KDM7A	1.23
ENSCHIG00000006309	2.14
TARS3	1.96
DDX11	-1.02
ENSCHIG00000024760	-2.18
B4GALNT1	-4.50
ENSCHIG00000005111	-10.77
SNORD49A	1.45
LGI4	-4.04
SYNPR	2.05
SLC39A5	1.33
GNMT	1.13
KCNC3	-1.86
ENSCHIG00000003716	3.64
SWT1	-1.15
FZD5	1.26
SLC18A2	-1.40
CAMK2B	8.71
ZG16	10.34
PCDH8	8.54
ENSCHIG00000004922	-1.11
SNORA44	1.48
ERBB3	2.18
CSF1R	1.53
OTOF	1.95
NRAP	-1.66
MPO	3.00

C7orf57	-1.43
AXDND1	-9.40
ENSCHIG00000001086	1.32
ATP6V1B1	-1.26
GPX2	-1.10
SNORD30	2.01
ARL6	-1.04
NMNAT3	-1.04
SNORD35B	2.00
ENSCHIG00000023039	-1.47
KCNE1	-1.58
VSTM5	-1.35
BAIAP2L2	3.32
ANGPT4	-1.84
SYT12	-1.32
KLHL33	-2.48
ENSCHIG00000009940	1.97
ENSCHIG00000024769	1.42
ENSCHIG00000015104	1.13
ENSCHIG00000025977	1.25
ENSCHIG00000026901	1.74
ENSCHIG00000006057	-11.40
TMEM132C	-1.32
PKLR	-2.74
ENSCHIG00000008140	11.06
PDE1B	-1.58
ABLIM2	-3.13
WNT9A	-1.85
ITGA9	-1.33
WNT11	1.16
Metazoa_SRP	1.73
ENSCHIG00000012142	-9.81
ENSCHIG00000002248	1.57
SNORA52	1.17
Metazoa_SRP	1.65
ENSCHIG00000025835	-12.42
ENSCHIG00000015134	1.95
IL20RB	1.29
AP3B2	-9.04
C7orf61	1.58
SNORD29	1.90
SH2D3A	-6.08
CCDC65	-1.33

SNCAIP	-1.05
ENSCHIG00000011258	-11.17
KLHL30	2.93
AQP3	-10.44
ERN2	3.47
ENSCHIG00000006914	11.58
ENSCHIG00000026724	2.36
ADRA2C	2.15
APBB1IP	-9.50
MYBPC1	-8.75
PTK2B	-1.70
ENSCHIG00000010711	-1.01
ENSCHIG00000013209	2.54
TMEM86B	1.02
GRID2	-1.70
ENSCHIG00000009222	-1.06
ARC	1.60
TRPM2	-8.43
ENSCHIG00000008842	2.01
SOSTDC1	8.70
TMEM198	-1.38
ENSCHIG00000014143	-1.45
CDH18	3.81
SNORD33	1.53
SEC16B	-3.26
ENSCHIG00000014623	-1.97
OCA2	-1.09
NPB	11.91
ACVRL1	-2.01
ACKR4	-1.16
GABRA4	-1.16
TENM4	-1.88
ELL3	1.19
COL8A2	-1.52
TTC29	-1.01
ENSCHIG00000013293	10.02
TCP11	-3.97
ENSCHIG00000002649	1.74
LRRC25	1.48
ENSCHIG00000007393	1.41
ENSCHIG00000005552	1.15
FAM20A	-1.33
REC8	1.48

NXPH4	1.94
TNFRSF13C	1.65
HKDC1	2.43
TAGLN3	1.76
ENSCHIG00000024072	-1.18
ENSCHIG00000005096	1.53
SNORD4A	1.12
LRRC9	-2.39
CLCA2	-8.77
ZNF296	2.03
ENSCHIG00000016045	-1.25
MYRFL	3.37
KCNN3	1.62
ENSCHIG00000009401	-1.80
PAX7	7.82
SNORD12C	1.71
GLI1	-2.80
ENSCHIG00000011376	3.28
HHLA2	-1.53
SFXN5	-1.09
ENSCHIG00000018753	9.76
SYTL3	-1.29
LRRIQ4	4.04
LAG3	1.11
ENSCHIG00000022972	11.93
TSHZ2	-1.01
PROB1	1.22
ENSCHIG00000017601	1.57
FUT1	-1.32
OPRD1	-10.10
PODNL1	-1.38
CA2	2.33
ENSCHIG00000009839	-1.26
ENSCHIG00000010477	2.12
PCSK4	-1.38
PHKG1	-1.23
CHODL	-1.83
STX11	-1.77
STING1	-1.05
FGF1	-1.11
PPARG	2.11
PSD2	1.25
NR1H4	-1.85

ATP1A3	2.14
NTNG2	-1.10
NCF4	-1.18
ABCC6	1.14
ENSCHIG00000013181	2.93
ENSCHIG00000021733	-1.26
ENSCHIG00000003847	-1.04
ENSCHIG00000013757	3.11
TNFSF13	1.79
RINL	3.27
EVA1A	-1.31
GFRA1	-2.56
ENSCHIG00000002545	-1.42
SNORA57	1.48
ENSCHIG00000020067	-1.77
CFAP58	-2.29
KCNJ14	1.40
SYCP2	2.99
POU4F1	1.93
ZNF804A	2.37
PARD6A	-1.60
CTSW	3.17
ENSCHIG00000011399	11.70
ENSCHIG00000026395	12.00
KCND3	3.70
OIT3	1.13
TMEM154	-2.61
GPR182	-2.31
RORC	-9.91
LTA	4.09
MAP6	1.23
ARMC2	-1.11
SIT1	7.35
ENSCHIG00000001391	9.26
SLC35F1	1.26
RAD51B	-1.05
GCG	3.91
ENSCHIG00000000245	-1.36
ECT2L	1.40
GATA2	2.28
GTF2A1L	-3.02
AMTN	-10.04
ENSCHIG00000008340	11.58

TACSTD2	1.33
PITX3	1.33
ENSCHIG00000008864	1.65
DKK1	2.21
ENSCHIG00000005546	8.85
ANKEF1	-1.15
SNORA24	1.18
SOAT2	2.24
TAS1R3	1.34
ENSCHIG00000017505	2.99
FLT4	-3.62
ZMYND15	-2.34
ENSCHIG00000005861	9.81
ENSCHIG00000007059	10.69
GABRR3	4.25
PCDHA4	-8.98
ENSCHIG00000007235	2.77
BHLHA15	10.99
MATK	4.76
ENSCHIG00000005313	1.05
ENSCHIG00000019523	-1.15
CAMK1D	-1.45
GPR68	2.08
TBXAS1	1.24
SNORA4	1.95
KCNJ2	9.68
TRIM6	-1.09
RGS10	-1.17
ENSCHIG00000019231	8.44
KLF1	9.91
SNORA67	1.05
ENSCHIG00000013608	-1.57
TLE7	10.05
ADGRF4	8.21
DISC1	-2.38
SEMA4A	8.90
ENSCHIG00000006459	1.34
ENSCHIG00000011862	11.04
ENSCHIG00000011371	-1.56
ENSCHIG00000002407	9.27
CYP2S1	-8.58
MACC1	3.49
CD37	1.40

CLDN9	4.08
SYTL1	-1.01
RASGEF1C	-1.13
PTH1R	-1.04
BTBD16	-9.96
NLRC5	4.99
FOXE3	5.29
BICDL2	9.39
ENSCHIG00000007567	7.45
ODAD4	-3.07
EHF	-1.44
COCH	9.10
DIRAS1	10.78
ENSCHIG00000011065	1.63
NTRK2	1.14
SYN2	-2.52
PIK3CD	-1.68
ABAT	-1.22
NEURL3	-3.38
HAPLN1	2.92
ENSCHIG00000007078	10.16
ENSCHIG00000020284	-9.19
CHRM4	-9.72
ENSCHIG00000022348	-1.01
CNGB3	7.31
FAM107A	-2.78
SLC1A3	1.11
ABCA10	-2.45
IRX5	3.40
ENSCHIG00000016185	-2.17
ENSCHIG00000000607	-2.72
IGF1	-1.04
ENSCHIG00000013663	-1.18
SNORA10	1.29
FAM167A	-1.21
FHAD1	2.78
NPAS4	1.01
BEGAIN	8.49
ENSCHIG00000007973	-10.90
SNORD5	1.26
SLC16A9	2.59
KCTD8	1.59
PAX1	9.90

IZUMO4	-1.04
AHRR	-1.48
SNORD101	2.23
RNF152	4.06
ENSCHIG00000024696	11.93
MERTK	-1.35
RELN	2.18
CLXN	-11.03
C12orf50	-9.91
KIF6	-1.21
NAALADL1	1.07
FBXL22	-10.24
NEK11	-1.22
MGLL	1.24
MKRN3	-9.53
SNORD17	1.63
ENSCHIG00000025947	9.02
RNF223	10.41
ABCC2	3.08
PTPRC	-1.14
ENSCHIG00000023504	-11.74
ENSCHIG00000006958	9.50
ENSCHIG00000001486	-1.81
CLDN6	1.25
SPAG4	-1.06
CYP2C81	-1.12
LRRC26	2.36
B3GNT7	-2.90
IMPA2	10.38
SNORA70	2.33
PCDH1	2.15
CECR2	1.26
C1orf141	2.16
SPATC1L	9.87
LRRN3	1.02
DOK2	7.74
CCL20	-3.59
SNORD100	1.79
ENSCHIG00000012186	-1.94
MMP25	2.50
ENSCHIG00000026709	2.74
SALL1	1.34
IQUB	1.31

CPNE7	4.12
TTC23L	-1.33
CCDC40	-2.25
ENSCHIG00000023381	1.52
SNORD28	2.09
LAT	3.53
SNORA53	1.15
LSAMP	-1.22
MRC1	2.84
ENSCHIG00000027096	-10.90
ENSCHIG00000009552	9.76
ENSCHIG00000006998	10.99
LRFN1	-1.30
TRPM3	-1.21
AVIL	1.00
ENSCHIG00000001094	-2.54
ABHD16B	-1.27
ENSCHIG00000010723	3.06
ENSCHIG00000020577	-1.97
PJVK	-2.06
ENSCHIG00000009670	-2.60
ALB	-7.43
PGLYRP4	9.95
ENSCHIG00000008653	1.06
ENSCHIG00000018774	2.42
ENSCHIG00000020715	-10.09
ENSCHIG00000007959	1.47
GANC	-1.37
LPCAT2	-9.30
FN3K	-1.03
KCNAB1	1.15
PTGDR	2.70
FOXA1	8.93
SRCIN1	2.15
ENSCHIG00000004839	-9.82
SNORD50B	1.17
STK32C	-1.18
RHOH	2.98
PDE4C	3.14
PTPRT	-2.06
CAMK1G	-1.12
GARIN1B	2.18
CACNA1B	7.61

ENSCHIG00000003431	1.30
ENSCHIG00000003392	-3.34
RASEF	2.21
ENSCHIG00000000691	6.69
EHHADH	-1.03
GPRIN1	-1.46
SLC4A5	-8.09
ENSCHIG00000007651	1.06
SLC25A34	1.64
CACNA2D2	2.51
ENSCHIG00000011039	3.67
C2orf81	-2.22
C9orf43	-1.08
BTN2A2	9.32
NPTX1	9.61
ENSCHIG00000009885	11.00
ADAM20	3.10
SPATC1	8.82
DAB1	9.16
B3GALT2	1.21
ENSCHIG00000018149	10.80
MYO7B	7.22
ENSCHIG00000005479	8.15
ENSCHIG00000011159	9.27
AVP	-10.71
ENSCHIG00000025410	9.54
BGLAP	2.84
PTGDR2	9.75
ST8SIA2	1.13
